# Breast cancer prediction using genome wide single nucleotide polymorphism data

**DOI:** 10.1186/1471-2105-14-S13-S3

**Published:** 2013-10-01

**Authors:** Mohsen Hajiloo, Babak Damavandi, Metanat HooshSadat, Farzad Sangi, John R Mackey, Carol E Cass, Russell Greiner, Sambasivarao Damaraju

**Affiliations:** 1 Department of Computing Science, University of Alberta, Edmonton, Alberta, Canada; 2 Alberta Innovates Centre for Machine Learning, University of Alberta, Edmonton, Alberta, Canada; 3 Department of Oncology, University of Alberta, Edmonton, Canada; 4 Department of Laboratory Medicine and Pathology, University of Alberta, Edmonton, Alberta, Canada; 5 PolyomX Program, Cross Cancer Institute, Alberta Health Services, Edmonton, Alberta, Canada

## Abstract

**Background:**

This paper introduces and applies a genome wide predictive study to learn a model that predicts whether a new subject will develop breast cancer or not, based on her SNP profile.

**Results:**

We first genotyped 696 female subjects (348 breast cancer cases and 348 apparently healthy controls), predominantly of Caucasian origin from Alberta, Canada using Affymetrix Human SNP 6.0 arrays. Then, we applied EIGENSTRAT population stratification correction method to remove 73 subjects not belonging to the Caucasian population. Then, we filtered any SNP that had any missing calls, whose genotype frequency was deviated from Hardy-Weinberg equilibrium, or whose minor allele frequency was less than 5%. Finally, we applied a combination of MeanDiff feature selection method and KNN learning method to this filtered dataset to produce a breast cancer prediction model. LOOCV accuracy of this classifier is 59.55%. Random permutation tests show that this result is significantly better than the baseline accuracy of 51.52%. Sensitivity analysis shows that the classifier is fairly robust to the number of MeanDiff-selected SNPs. External validation on the CGEMS breast cancer dataset, the only other publicly available breast cancer dataset, shows that this combination of MeanDiff and KNN leads to a LOOCV accuracy of 60.25%, which is significantly better than its baseline of 50.06%. We then considered a dozen different combinations of feature selection and learning method, but found that none of these combinations produces a better predictive model than our model. We also considered various biological feature selection methods like selecting SNPs reported in recent genome wide association studies to be associated with breast cancer, selecting SNPs in genes associated with KEGG cancer pathways, or selecting SNPs associated with breast cancer in the F-SNP database to produce predictive models, but again found that none of these models achieved accuracy better than baseline.

**Conclusions:**

We anticipate producing more accurate breast cancer prediction models by recruiting more study subjects, providing more accurate labelling of phenotypes (to accommodate the heterogeneity of breast cancer), measuring other genomic alterations such as point mutations and copy number variations, and incorporating non-genetic information about subjects such as environmental and lifestyle factors.

## Background

Cancer is a complex disease, characterized by multiple molecular alterations triggered by genetic, environmental and lifestyle effects. Cancer cells typically accumulate alterations disrupting the cell's life cycle of growth, proliferation, and death [1]. Genomic changes that can eventually lead to cancer include mutations (<1% in frequency), single nucleotide polymorphisms (SNPs, >1% in frequency), insertion and deletion polymorphisms and structural changes in chromosomes. SNPs are the most common type of inherited genomic variation and recent advances in high-throughput technologies have led to whole-genome SNP arrays; datasets of such profiles over many subjects provide a valuable way to discover the relationship between SNPs and diseases such as cancer [2].

A genome wide association study (GWAS) compares the SNP profiles, over a wide range of SNPs, of two groups of participants: e.g., people with the disease (cases) versus people without the disease (controls). Each individual SNP whose values are significantly different between these groups (typically based on chi-square test between the values observed for the two groups) is said to be *associated *with the disease [3]. Of course, the resulting associated SNPs even those with high statistical significance using genome-wide corrections for multiple hypothesis testing are at best proxies for truly causal information, which can only be obtained through further deep sequencing of the associated loci and well-designed appropriate wet-lab studies. The database of Genotypes and Phenotypes (dbGaP) archives and distributes the results of studies that have investigated the interaction of a genotype and phenotype in GWASs [4]. However, while GWASs can help the researchers better understand diseases, genes and pathways, they are not designed to predict whether a currently undiagnosed subject is likely to develop the disease.

This paper introduces Genome Wide Predictive Studies (GWPSs), which take the same input as a GWAS (the SNP arrays for a set of subjects, each labelled as a case or a control) but outputs a *classification model *that can be used later to predict the class label of a previously undiagnosed person, based on his/her SNP profile. The field of machine learning provides a variety of statistical, probabilistic and optimization techniques that allow computers to learn such classifiers from these datasets of labelled patients. Machine learning has been applied successfully in many areas of biology and medicine, often to produce effective predictors. Baldi and Brunak [5], Larranga et al. [6], Tarca et al. [7], Cruz and Wishart [8] each surveyed various applications of machine learning in biology, including gene finding [9], eukaryote promoter recognition [10], protein structure prediction [11], pattern recognition in microarrays [12], gene regulatory response prediction [13], protein/gene identification in text [14], and gene expression microarray based cancer diagnosis and prognosis [8]. We consider a way to learn a predictor ("who has breast cancer?"), for a dataset that specifies all available SNPs about each subject.

Our "genome wide" approach differs from research that attempts to learn predictors from only a pre-defined set of candidate SNPs. As an example of such a candidate SNP study, Listgarten et al. [15] applied a machine learning tool (support vector machine, SVM) to a pre-defined set of 98 SNPs, distributed over 45 genes of potential relevance to breast cancer, to develop a predictive model with 63% accuracy for predicting breast cancer. Ban et al. [16] applied a SVM to analyze 408 SNPs in 87 genes involved in type 2 diabetes (T2D) related pathways, and achieved 65% accuracy in T2D disease prediction. Wei et al. [17] studied type 1 diabetes (T1D) and reported 84% area under curve (AUC) using an SVM.

Our approach also differs from the conventional risk modeling/prediction studies. Those studies also begin with a small set of pre-defined features: they first sort the training subjects into a small set of bins, based on the values of these features e.g., the Gail model uses 7 features and record the percentage in each bin with the phenotype (here breast cancer) [18,19]. Afterwards, to estimate the risk a new subject will face, this tool uses the subject's values for those relevant features to sort that subject into the proper bin, and returns the associated probability (called risk). Hence this approach bases its assessment on only a small number of pre-specified features. Note this might not be sufficient to usefully characterize the subjects, especially if the hand-picked features are not adequate. On the other hand, our machine learning (ML) approach lets the data dictate on the possible combination of features that are relevant. (While the ML model described in this paper returns a specific prediction for the individual here breast cancer or not there are other ML models that will return the probability that the individual will have the disease P(disease | feature_values), which is basically risk). Our general goal is to develop a tool to help screen women, by predicting which of the apparently healthy subjects sampled in a population will eventually develop breast cancer. This cannot be done by gene expression-based microarray analyses, as those results require biopsies of tissues from organs or tumours, which means they are only relevant to individuals with suspect tissues; hence they are not effective at identifying individuals at risk in a general population, before the onset of the disease, and so cannot be used for our early detection. The standard breast cancer risk assessment model (the Gail model [18,19], described above) is designed to help with early detection; however, it has only limited clinical value. Note that researchers recently extended this Gail model by including 7 or 10 SNPs associated with breast cancer susceptibility (from GWASs); however, this led to only marginally improved accuracy [20,21].

This paper presents a method to learn, from a dataset containing genome-wide SNPs of a cohort of subjects (cases and controls), a classifier that can predict whether a new subject is predisposed to the phenotype of breast cancer. (Note this classifier differs from the Gail model, as it can assign each individual subject to a label, potentially based on all of the features describing that subject.) We describe the challenges of addressing this high-dimensional data and show that a learner is capable of producing a classifier that can identify, with 59.55% accuracy, whether the subject has breast cancer, based only on her SNP profile. While this might not be clinically relevant, this performance is statistically significantly better than the baseline (of just predicting the majority class), which demonstrates that (1) there is information relevant to breast cancer in a patient's SNP values (note our method uses only SNPs, but not demographic data, nor other environmental data) and (2) that today's machine learning tools are capable of finding this important information.

## Methods

**Figure 1:**
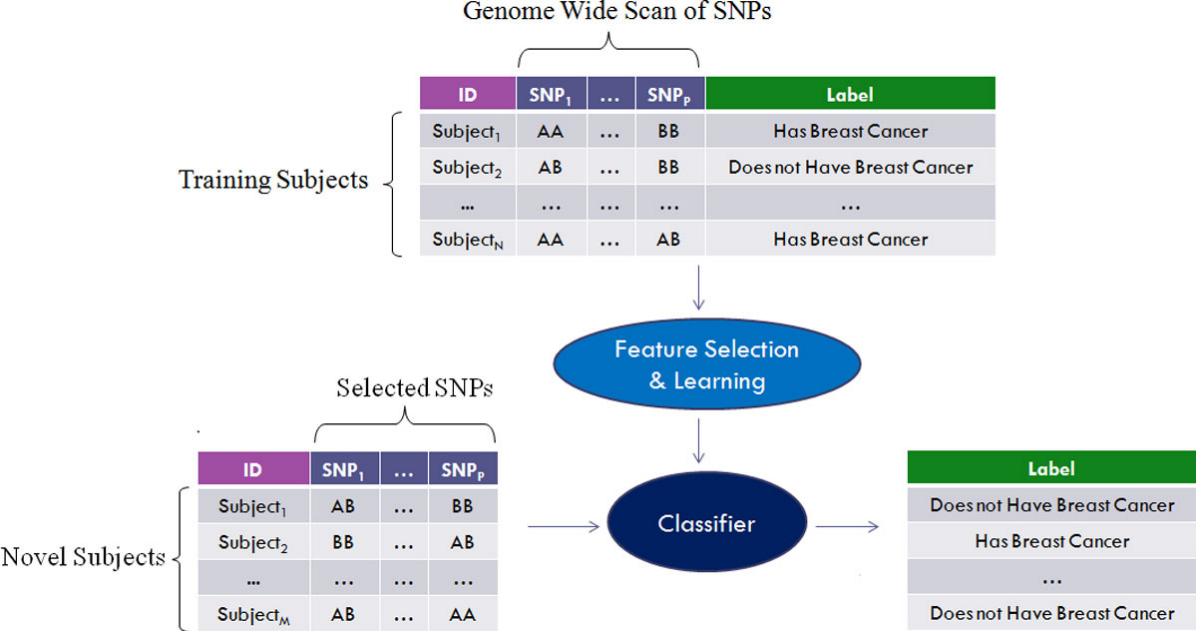
**A schema of a genome wide predictive study (GWPS)**. Given a labelled training dataset of subjects each described by a genome wide scan of SNPs, feature selection and learning methods are applied to learn a classifier that can predict the labels of a set of novel subjects.

In general, a Genome Wide Predictive Study (GWPS) takes as input the SNP profiles of a set of N individuals (including both cases and controls) and outputs a classifier, which can later be used to predict the class label of a new individual, based on his/her SNP profile; see Figure 1. Here, we used a dataset of N = 696 subjects including 348 breast cancer cases (late onset of disease, i.e., of sporadic nature) and 348 controls (disease free at the time of recruitment and with no family history of breast cancer), accessed from a previous study on sporadic breast cancer wherein breast cancer predisposition in women is not related to mutations in the known high penetrance breast cancer genes (eg, BRCA) nor other genes of moderate penetrance, described in earlier studies [22]. Germline DNA was isolated from peripheral blood lymphocytes. Genotyping profiles were generated using Affymetrix Human SNP 6.0 array platform (906,600 SNPs on each array). The study subjects provided informed consent and the study was approved by the Alberta Cancer Research Ethics Committee of the Alberta Health Services. Following probe labelling, hybridization and scanning, population stratification correction using EIGENSTRAT removed 73 subjects (46 cases and 27 controls) that did not co-cluster with Hapmap II Caucasian subjects, which left 623 Caucasian subjects (302 cases and 321 controls) [23]. After that, the dataset was filtered by removing any SNP (1) that had any missing calls, (2) whose genotype frequency deviated from Hardy-Weinberg equilibrium (nominal p-value <0.001 in controls) or (3) whose minor allele frequency were less than 5% (>5% frequency considered as common variants); this left a total number of 506,836 SNPs for analysis. For each SNP, we represented wild type homozygous, heterozygous and variant homozygous by 1, 2, and 3 respectively.

A trivial classifier, which just predicts the majority class (here control), will be 321/623 = 51.52% accurate. The challenge is producing a classifier that uses subject SNP data to produce predictions that are significantly more accurate. In particular, we explored tools that use the given labelled dataset to find the patterns that identify breast cancer (i.e., case versus control). Fortunately, the field of machine learning (ML) provides many such learning algorithms, each of which takes as input a labelled dataset, and returns a classifier. These systems typically work best when there are a relatively small number of features typically dozens to hundreds but they tend to work poorly in our situation, with over half-a-million features; here, they will often over-fit [24]: that is, do very well on the training data as they find ways to fit the details of this sample, but in a way that does not work well on the subjects that were not part of the training dataset. Note that our goal is to correctly classify such novel (that is, currently-undiagnosed) subjects. We therefore apply a pre-processing step to first reduce the dimensionality of the data, by autonomously identifying a subset of the most relevant SNPs (features). We then give this reduced dataset to a learning algorithm, which produces a classifier [25]. We later discuss how to evaluate the classifier produced by this "feature selection + learning" system.

### Feature Selection

In our analysis, as we expect only a subset of the SNPs to be relevant to our prediction task, we focused on ways to select such a small subset of the features. In general, this involves identifying the features that have the highest score based on some criteria (which we hope corresponds to being most relevant to the classification task). In this study, we used the MeanDiff feature selection method, which first sorts the SNPs based on their respective MeanDiff values, which is the absolute value of the difference between mean values of this SNP over the cases and the controls:

(1) $$\text{MeanDiff}\left(\text{SN}{\text{P}}_{\text{i}},\text{D}\right)=\;|\mu \left(\text{i},\text{C}\right)-\mu \left(\text{i},\text{H}\right)|$$

over the dataset D = C ∪ H where C is the set of subjects known to have cancer (each labelled as case) and H is the remaining healthy subjects (each labelled as control), and using Expr(i,j) as the value of the i'th SNP of subject j, $\mu \left(i,H\right)=\frac{1}{\left|H\right|}\sum_{j\in H}Expr\left(i,j\right)$ is the mean value of the i'th SNP over the subset H (the controls) and $\mu \left(i,C\right)=\frac{1}{\left|C\right|}\sum_{j\in C}Expr\left(i,j\right)$ is the mean value of the i'th SNP over the subset C (the cases). Note this MeanDiff(SNP_i_, D) score will be 0 when SNP_i _is irrelevant and presumably larger for SNPs that are more relevant to our prediction task. Here, we decided to use the m = 500 SNPs with the largest MeanDiff values; see the summary information of these top 500 MeanDiff selected SNPs in Additional file 1: Appendix1.

### Learning

To build a classifier, we use the very simple learning algorithm, K-Nearest Neighbors (KNN), which simply stores the (reduced) profiles for all of the training data [26]. To classify a new subject *p*, this classifier determines *p*'s k nearest neighbors, and then assigns p the majority vote. (So if k = 5, and *p*'s 5 closest neighbors include 4 controls and 1 case, then this classifier assigns *p *as control). Of course, we need to define distances to determine the nearest neighbors. As we are representing each patient as a m-tuple of the SNP values, we define the distance between two individuals *p *= [p_1_, ..., p_m_] and *q *= [q_1_, ..., q_m_] as the square of the Euclidean distance (aka L2 distance) as shown below.

(2) $$\text{d}\left(\text{p},\text{q}\right)=\sum_{\text{i}=1}^{\text{m}}{\left({\text{p}}_{\text{i}}-{\text{q}}_{\text{i}}\right)}^{2}$$

### Learning Parameter Selection

Notice the KNN learning algorithm requires us to specify how many neighbors to consider the k mentioned above. Which value should we use i.e., should we use k = 1 (i.e., consider only the single nearest neighbor), or k = 3 or k = 5 or...? It is tempting to set k by: running 1-NN on the data, then determining the apparent error (using leave-one-out cross validation see below), then computing the error associated with 3-NN, then 5-NN, and so forth; and finally selecting the value k ∈ {1, 3, 5, 7} that produces the smallest error. Unfortunately, this would mean finding a relevant parameter based on its score on the full set of training data, which corresponds to testing on the training data. That is, the k-value that optimizes that score might not be the one that produces the best performance on novel subjects, as the value determined in this fashion can lead to serious over-fitting.

We therefore need a more elaborate method, BestKNN, to determine the appropriate values for this parameter. Here, BestKNN first divides the training data into r = 10 disjoint subsets, D = D_1 _∪... ∪D_r_, then for each i = 1..r, defines D_-i_=D - D_i _as the complement of D_i_, and lets C_i1 _be the 1-NN classifier that is trained on D_-i_. For each i, the C_i1 _classifier uses the m SNPs that have the best MeanDiff(., D_-i_) scores, based on the D_-i _dataset. As D_-i _is different from D_-j _when i≠j, the m SNPs used by C_i1 _will typically be different from the m SNPs used for C_j1_. BestKNN then computes the accuracy, acc(C_i1_, D_i_), of this C_i1 _classifier over D_i _ie, over data that it was not trained on. It then computes the average accuracy over all r different folds, $score\left(1,D\right)=\frac{1}{r}\sum_{i=1}^{r}acc\left(C_{i1},D_{i}\right)$ which is an estimate of how well 1-NN would work over the complete dataset D. BestKNN similarly computes score (3,D) based on 3-NN, and score(5,D), etc., for k∈{1, 3, 5, 7}, then uses the high-watermark as the appropriate value of k. Here, using r = 10 folds, it found k* =7 worked best for our dataset (note this requires computing the top m SNPs, then running the resulting KNN, for 4×10 different datasets; the only purpose of all of this work is to find this k* value). BestKNN then defines the final classifier based on the top m SNPs over the entire dataset, using this specific k* =7 value.

### Evaluation

The next challenge is estimating the quality of the classifier, C_623 _= BestKNN(D_623_) the classifier produced by running BestKNN (which involves the m best MeanDiff SNPs), on our 623 subject cohort D_623_. Here we use two strategies to evaluate our classification algorithm: (1) by using Leave-One-Out Cross Validation (LOOCV) strategy and (2) by using an external hold-out (validation) dataset.

First, we use the LOOCV strategy, which first runs the BestKNN algorithm to produce a classifier based on N-1 = 622 training subjects (of the dataset with N=|D|=623 subjects), which is then tested on the 1 remaining subject. We ran these processes N times, so that every subject is used one time as the test dataset. We estimate the true accuracy of C_623 _as the percentage of correctly classified subjects, over these 623 folds. Producing this estimate means running all of BestKNN 623 more times which, recall, each involves computing the top m SNPs for 40+1 different configurations. Some earlier researchers mistakenly ran their feature-selection process over the entire dataset D, and then committed to these features for all folds of the cross-validation process. Unfortunately, this gives inaccurate (overly optimistic) estimates [27-29]. On our task, we found that this incorrect process suggests that the resulting classifier has an apparent accuracy of over 90% -- which is considerably above its true accuracy of around 60% (see below).

Second, we used an external validation dataset of 2287 subjects (1145 breast cancer cases and 1142 controls) from the Cancer Genetic Markers of Susceptibility (CGEMS) breast cancer project [30]. Genotyping profiles for these subjects were generated using Illumina HumanHap550 (I5) array platform (555,352 SNPs on the array).To date, this is the only publicly available dataset related to a genome wide association study of breast cancer, which is on Caucasian population set.

## Results

**Figure 2:**
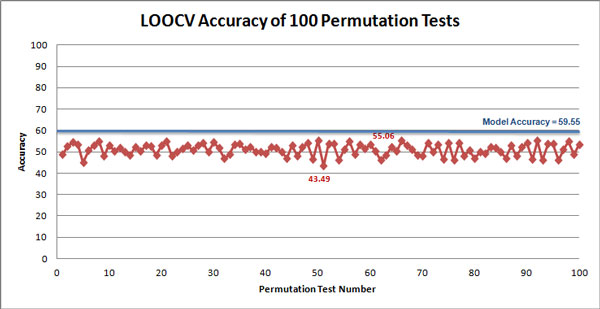
**Accuracy of a hundred "Permute, Learn, and Evaluate" Instances**. The accuracies of 100 random permutation tests. We see that none of these accuracies exceeded the 59.55% accuracy of our model. This means that our result is significantly better than the baseline, with a confidence of more than 99%.

**Figure 3:**
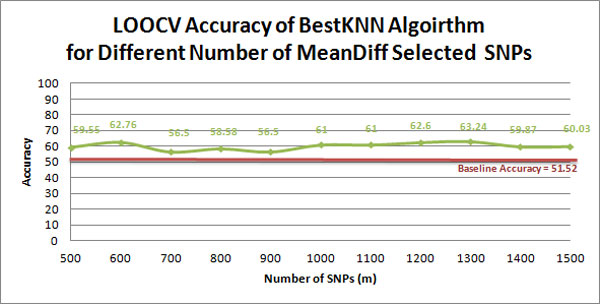
**Accuracy of the BestKNN algorithm for different numbers of MeanDiff selected SNPs**. Accuracy of the classifiers built using BestKNN on sets of SNPs with the top {500, 600, ..., 1500} MeanDiff scores. This suggests that our model is fairly robust to the number of MeanDiff-selected SNPs, when selecting more than 500 SNPs.

Table 1 provides the confusion matrix of actual versus predicted labels given by the classification model built using BestKNN, over the specified dataset. Our LOOCV estimates the accuracy of this model to be 59.55%; with precision 50.40%, recall/sensitivity 61.92%, and specificity 57.32%. To test if this result is significantly more accurate than the baseline of 51.52%, we applied a permutation test [31]. Here, we permuted the labels in the original dataset randomly, which should destroy any signal relating the SNPs to the cancer/no-cancer phenotype. We then ran the BestKNN to build new classifiers on this new dataset, and ran the LOOCV process to estimate the accuracy of the new model. We repeated this "permute, learn, evaluate" process over 100 permutations. As presented in Figure 2, none of these accuracies (of the 100 models built over randomly permuted labelled datasets) exceeded the 59.55% accuracy of our model. This suggests that our result is significantly better than the baseline, with a confidence of more than 1 1/100 = 0.99 ie, the associated p-value is p<0.01. Figure 3, which provides the LOOCV accuracy of the classification model built using BestKNN on sets of SNPs with the top {500, 600, ..., 1500} MeanDiff scores, suggest our model is fairly robust to the number of MeanDiff selected SNPs, when selecting more than 500 SNPs.

**Table 1:** Confusion matrix for comparison of actual and predicted labels on 623 breast cancer study subjects

		Predicted Label	
		
		Case	Control
**Actual Label**	**Case**	187 (TP)	115 (FP)
	
	**Control**	137 (FN)	184 (TN)

Accuracy = (TP+TN)/(TP+FP+TN+FN)=59.55%; Precision = TP/(TP+FP)=50.40%;

Recall/Sensitivity = TP/(TP+FN)=61.92%; Specificity = TN/(TN+FP)=57.32%.

To test the effectiveness of our approach, we next explored ways to apply it to other datasets. The standard approach involves running the resulting classifiers on another dataset, whose subjects include values for the same set of features and are labeled with the same phenotypes. Unfortunately, there are no other public datasets for this phenotype that use the same Affymetrix Human SNP 6.0 array Platform. We did, however, consider applying our C_623 _= BestKNN(D_623_) classifier on the CGEMS breast cancer dataset that includes 1145 breast cancer cases and 1142 controls genotyped on the Illumina I5 array platform. Unfortunately, due to this difference between the platforms, this dataset includes only 101 SNPs in common with the m = 500 SNPs used by C_623_. As this meant the CGEMS data was missing ~80% of the SNP values used by C_623_, we obviously could not apply C_623 _directly on this dataset. As this CGEMS breast cancer dataset is the only available genome-wide dataset on Caucasian population, we therefore had to design another experiment to evaluate our approach, based on the MeanDiff_500_+BestKNN learning method. Here, we used the same MeanDiff_500_+BestKNN algorithm, but here trained this method over D_2287_, the 2287 subjects of CGEMS breast cancer dataset. We again evaluated the performance of this learned model using the LOOCV method. Table 2 shows the estimated accuracy of this learning algorithm on this external validation dataset, BestKNN(D_2287_), is 60.25% (which is significantly better than the baseline of 50.06%), with precision 60.44%, recall/sensitivity 59.65%, and specificity 60.86%. This confirms that our approach and algorithm, is reproducible, as this exact system works effectively on a second, very different breast cancer dataset. Notice others have used the same validation approach; see [32].

**Table 2:** Confusion matrix for comparison of actual and predicted labels on 2287 CGEMS breast cancer dataset

		Predicted Label	
		
		Case	Control
**Actual Label**	**Case**	683 (TP)	462 (FP)
	
	**Control**	447 (FN)	695 (FN)

Accuracy = (TP+TN)/(TP+FP+TN+FN)=60.25%; Precision = TP/(TP+FP)= 60.44%;

Recall/Sensitivity = TP/(TP+FN)=59.65%; Specificity = TN/(TN+FP)=60.86%.

Hoping to further improve these results, we explored several techniques both biologically naïve and informed for both selecting features and for building the classifier itself. To select features, we considered biologically naïve methods such as information gain [33], minimum redundancy maximum relevance (mRMR) [34] and principal component analysis (PCA) [35]. We also applied other biologically naïve learning algorithms, including decision trees [33], and support vector machines (with RBF kernel) [36]. In all, we tried dozens of different combinations of the learning and feature selection algorithms (each with its own range of parameters values) each of which proved to be computationally intensive (several CPU days). Table 3 shows the accuracy of each of these combinations. Here, we see that none of these combinations are more accurate than our suggested combination of MeanDiff_500 _feature selection and BestKNN learning (59.55%); indeed, several do not even beat the baseline of 51.52%.

**Table 3:** Accuracy of a dozen of different combinations of feature selection and learning methods

		Feature Selection Methods			
		
		Information Gain	MeanDiff	mRMR	PCA
**Learning Methods**	**Decision Tree**	50.88%	52.06%	51.20%	51.69%
	
	**KNN**	56.17%	58.71%	57.78%	51.36%
	
	**SVM-RBF**	55.37%	57.30%	56.18%	51.84%

10-fold cross validation accuracies of combination of 4 feature selection methods and 3 learning methods shows that none of these combinations are more accurate than our suggested combination of MeanDiff_500 _feature selection and BestKNN learning (59.55%); indeed, several do not even beat the baseline of 51.52%.

We also used biological information related to cancer to inform feature selection i.e., use SNPs known to be relevant to breast cancer, rather than our biologically-naïve MeanDiff method: First, we considered the 28 SNPs identified by recent GWASs as being highly associated with breast cancer (see Table 4; [30,37-43]). We trained KNN over the 623 subjects, but using only these 28 SNPs. Unfortunately the LOOCV of this classifier was just baseline, indicating that the SNPs that appear to be the most associated content with breast cancer are not sufficient to produce an effective classifier. Indeed, none of those 28 SNPs appear in the top 500 that MeanDiff selected. While different studies often identify different SNPs as significant, biological pathways seem much more stable, in that certain pathways are identified across multiple studies. This motivated us to try using only the 12,858 SNPs associated with genes of the KEGG's cancer pathways [44] recognized as hallmarks of cancer [1]; unfortunately, the classifier based on these features also did not perform better than baseline. Finally, we built a classifier using only the 1,661 SNPs associated with breast cancer in the F-SNP database [45]; this too had just baseline accuracy. These negative results show that the obvious approach of first using prior biological information to identify SNPs, and then learning a classifier using only those SNPs, does not seem to work here.

**Table 4:** List of breast cancer associated SNPs reported by recent genome wide association studies

dbSNP ID	Gene	Reference
rs2981579	FGFR2	Hunter et al., 2007 [30]

rs2420946	FGFR2	Hunter et al., 2007 [30]

rs11200014	FGFR2	Hunter et al., 2007 [30]

rs7696175	TLR1/TLR6	Hunter et al., 2007 [30]

rs17157903	RELN	Hunter et al., 2007 [30]

rs1219648	FGFR2	Hunter et al., 2007 [30]

rs3803662	TNRC9/LOC643714	Easton et al., 2007 [37]

rs889312	MAP3K1	Easton et al., 2007 [37]

rs13281615	8q	Easton et al., 2007 [37]

rs3817198	LSP1	Easton et al., 2007 [37]

rs2981582	FGFR2	Easton et al., 2007 [37]

rs2075555	COL1A1	Murabito et al., 2007 [38]

rs1978503	FLJ45743	Murabito et al., 2007 [38]

rs1926657	ABCC4	Murabito et al., 2007 [38]

rs13387042	2q35	Stacey et al., 2007 [39]

rs3012642	PHKA/HDAC8	Gold et al., 2008 [40]

rs7203563	A2BP1	Gold et al., 2008 [40]

rs6569479	ECHDC1/RNF146	Gold et al., 2008 [40]

rs2180341	ECHDC1/RNF146	Gold et al., 2008 [40]

rs6569480	ECHDC1/RNF146	Gold et al., 2008 [40]

rs4415084	5p12	Stacey et al., 2008 [41]

rs10941679	5p12	Stacey et al., 2008 [41]

rs2067980	MRPS30	Thomas et al., 2008 [42]

rs7716600	MRPS30	Thomas et al., 2008 [42]

rs11249433	1p11.2	Thomas et al., 2008 [42]

rs999737	RAD51L1	Thomas et al., 2008 [42]

rs4973768	SLC4A7	Ahmed et al., 2009 [43]

rs6504950	STXBP4	Ahmed et al., 2009 [43]

28 SNPs identified by the 8 recent genome wide association studies on breast cancer. The accuracy of the classifier learned over these 28 genotyped SNPs was not better than the baseline of 51.52%.

## Discussion

Our study confirms that SNPs do carry information related to breast cancer genetic susceptibility, and that GWPSs are a promising tool for decoding and exploiting this information. While this approach is theoretically applicable for studying other cancer types and diseases, we list below some of the potential limitations that may make it difficult to produce more accurate prediction models, for breast cancer or other diseases:

**Small sample size vs. large feature size: **As noted earlier, as the number of subjects in this study is significantly less than the number of SNPs (a few hundred instances versus half a million features), we face high-dimensionality problem, which can cause the learning systems to over-fit i.e., produce models that perform well on the training subjects but relatively poorly on new subjects distinct from those used for training. Two categories of techniques that attempt to tackle high-dimensionality are feature selection and sample integration. This report shows feature selection produces a classifier whose accuracy is significantly above baseline. Sample integration involves increasing the number of subjects in the study by either collecting more instances or by combining the dataset with other existing datasets, perhaps from different laboratories. However, there are still many significant challenges here, including dealing with batch effects [46].

**Breast cancer heterogeneity: **Breast cancer is biologically heterogeneous: current molecular classifications based on transcriptome-wide analysis, clinical determinations of steroid hormone receptor (like ER) status, human epidermal growth factor receptor 2 (HER2) status, or proliferation rate status (PR), all suggest a minimum of four distinct biological subtypes [47]. Our current dataset ignores the differences by merging these different sub-classes into the single label: case. We might be able to produce a more accurate predictor if we employed more detailed labelling of sub-cases, to produce a classifier that could map each subject to a molecular subtype. However, as our dataset is relatively small, further stratification of cases into subtypes of breast cancer might add to the high-dimensionality problem.

**SNPs are only one form of genomic alterations**: While this study considered only SNPs, there are also many other heritable genetic factors including mutations, copy number variations (CNVs), and other chromosomal changes. We believe that augmenting the SNP data with additional genetic information, such as insertion/deletion polymorphisms and CNVs, could lead to more accurate breast cancer predictive models. Of course, as this means using yet more features, this could also increase the risk of over-fitting.

**Breast cancer is also influenced by non-genetic factors**: Heritable factors are only part of the issue: while they play a major role in monogenic diseases such as haemophilia, diseases such as tuberculosis and lung cancer have a very high environmental and life style component, meaning genetic component contributes only a small amount to overall risk. Indeed, for many of diseases, the genetic component accounts for only 30-60% of the risk, with the remaining risk due to environmental and life style risk factors. There are many factors that contribute to developing breast cancer, in addition to heritable (DNA based) changes. The major environmental and lifestyle risk factors include age, estrogen exposure (from endogenous and exogenous sources), smoking, radiation exposure, obesity, and lifestyle in general [48]. As the breast cancer predictive model presented here used only germline DNA, it did not incorporate any of these non-genetic variables. We anticipate better results from a comprehensive model that includes both genetic and non-genetic factors.

## Conclusions

We present a genome wide predictive study as a way to understand, and effectively use, data from multiple single nucleotide polymorphisms. We first contrast this approach with the more standard associative studies, connecting this predictive approach directly with screening and personalized health care. We also show that it differs from the risk model (such as Gail) as our model can involve a large number of characteristics for each patient (here, hundreds of SNPs).

Our studies confirmed the feasibility of predicting breast cancer susceptibility from genome wide analysis of SNPs, by presenting a learning model that first uses the MeanDiff feature selection technique to identify the best subset of (m = 500) SNPs from the over-500K SNPs of the original dataset, then used k-nearest neighbour (with the k learned using an appropriate algorithm) as the classifier over these SNPs. Leave- one-out cross validation estimates the prediction accuracy of this proposed method to be 59.55%. A random permutation test indicated that this result is significantly better than the baseline predictor (p < 0.01). Sensitivity analysis on performance of our classifier showed that our model is robust to the number of MeanDiff-selected SNPs. We externally validated our learning algorithm using 2287 subjects from the CGEMS breast cancer dataset; this again produced a classifier whose LOOCV accuracy was significantly better than the baseline, which shows the reproducibility of our combination of MeanDiff and BestKNN in breast cancer prediction.

To better understand the challenge of this dataset, we systematically explored a large variety of other feature selection and learning algorithms. We found that none of the biologically naïve approaches to feature selection worked as well as our MeanDiff. We also considered many biologically-informed methods to select SNPs using SNPs reported in the literature to be associated with breast cancer, SNPs associated with genes of KEGG's cancer pathways, and SNPs associated with breast cancer in the F-SNP database. However, those SNPs produced classifiers that were not even better than baseline. These negative findings suggest the challenge of our task, and of the importance of findings of our study.

We also identified several limitations that may hinder a more accurate predictive model for breast cancer susceptibility. Sporadic breast cancer is a heterogeneous phenotype, which is also heavily influenced by environmental factors. Moreover, while our study does involve 623 samples, this is small relative to the number of features (SNPs) from a whole genome scan; we expect to achieve yet better results given a larger sample sizes. Furthermore, we anticipate developing better predictive models by incorporating other information both other genetic information (such as point mutations, copy number variations, and other structural chromosome changes using next generation sequencing) as well as environmental and lifestyle factors. The fact that our study produced statistically significant results, despite these limitations, demonstrates the potential of this machine learning approach in this context of screening, and of personalized patient care.

## Competing interests

The authors declare that they have no competing interests.

## Authors' contributions

MH designed and implemented the experiments and drafted the manuscript; BD, MHS, and FS helped running preliminary experiments; JRM provided insights from clinical oncology; CEC and SD as investigators on the Canadian Breast Cancer Foundation (CBCF) Tumor Bank in Alberta provided access to clinical data; RG participated in the design of experiments and manuscript edits; SD as the principal investigator of the whole genome breast cancer studies, offered data, provided suggestions during the course of experiments and edited the manuscript. All authors read and approved the final manuscript.

## Supplementary Material

Additional file 1 **Appendix1**. Summary information of the top 500 MeanDiff selected SNPs. Click here for file

## Declarations

RG provided the cost for the publication of this article.

This article has been published as part of BMC Bioinformatics Volume 14 Supplement 13, 2013: Selected articles from the 9th Annual Biotechnology and Bioinformatics Symposium (BIOT 2012). The full contents of the supplement are available online at http://www.biomedcentral.com/bmcbioinformatics/supplements/14/S13
